# ﻿A new species of *Docosia* Winnertz, 1864 and new records of fungus gnats (Diptera, Bolitophilidae, Keroplatidae and Mycetophilidae) from North Africa

**DOI:** 10.3897/zookeys.1233.130502

**Published:** 2025-04-07

**Authors:** Mohamed Amin El Mouden, Peter J. Chandler, Imane Saidoun, Ouafaa Driauach, Boutaïna Belqat

**Affiliations:** 1 LESCB URL/CNRST N 18, FS, Abdelmalek Essaâdi University, Tétouan, Morocco Abdelmalek Essadi University Tétouan Morocco; 2 606B Berryfield Lane, Melksham, Wilts SN12 6EL, UK Unaffiliated Wilts United Kingdom; 3 Biotechnology, Environmental Technology and Valorization of Bio-Resources Team, Department of Biology, Laboratory of Research and Development in Engineering Sciences Faculty of Sciences and Techniques Al-Hoceima, Abdelmalek Essaâdi University, Tétouan, Morocco Abdelmalek Essaâdi University Tétouan Morocco

**Keywords:** Biodiversity, caves, description, distribution, Middle Atlas, Morocco, National Park of Tazekka, taxonomy

## Abstract

A new species of Mycetophilidae in the genus *Docosia* Winnertz, 1864 is described, *Docosiatazekkae* Chandler, El Mouden & Belqat, **sp. nov.**, with the addition of eighteen new records of fungus gnats to the fauna of North Africa. Most of the new taxa of fungus gnats are found in Morocco, especially in the National Park of Tazekka, where a total of 69 species are recorded for the first time, thus enriching the fauna of these gnats in the Middle Atlas region; the greatest species richness is observed in caves. All these findings attest to the high biodiversity of the fungus gnats in Morocco, which, among the North African countries, has the best-known fauna.

## ﻿Introduction

Fungus gnats are a highly diverse group of Diptera. Traditionally they include six families in the superfamily Sciaroidea: Bolitophilidae, Diadocidiidae, Ditomyiidae, and Keroplatidae, which were classified earlier as subfamilies of Mycetophilidae (Edwards 1925), before being later recognised as independent families (Väisänen 1984b; Matile 1990; Hippa and Vilkamaa 2005). The fungus gnats group encompasses also the Mycetophilidae and Rangomaramidae families, however, the latter’s composition remains unclear. The fungus gnats include approximately 1500 species in the Palaearctic region alone (Søli et al. 2000) and more than 5000 species distributed worldwide (Pape et al. 2011). As for their morphology, adults are generally recognised by their hump-backed appearance, stout and elongated coxae and well-developed tibial spurs. According to Chandler (1999, 2022), all fungus gnat families are known to inhabit older, well-established forests and wooded biotopes rich in mature and decaying trees. Most species develop in fungi or decaying wood. Their larvae feed on decaying plant material, fungal mycelia in rotten wood, mushrooms, and in some special cases, on some bryophytes or are predaceous on other insects (Vockeroth 1981; Søli et al. 2000; Jakovlev 2011). The more humid the forests, the more they are favoured, and adults are usually most abundant in sheltered habitats (such as caves or shady biotopes) and near streams (Chandler 1999, 2022). Despite being primarily forest dwellers, fungus gnats can be recorded in various ecosystems, usually associated with fungal habitats (Kerr 2008); fewer species are therefore found in warm, dry and more open biotopes (Chandler 1999). Adults of fungus gnats often occur in large numbers and play important roles, especially in the forest environment food web (Kerr 2008).

The fungus gnat fauna of North Africa was hitherto represented by 114 species from 41 genera and three families: Bolitophilidae (one species from one genus), Keroplatidae (19 species from 10 genera) and Mycetophilidae with 94 species from 30 genera (Becker 1907; Enderlein 1913; Lundström 1916; Madwar 1935; Burghele-Balacesco 1967, 1972; Laštovka and Matile 1974; Matile 1977; Gagné 1981; Väisänen 1984a, 1984b; Bechev 1989; Caspers 1991; Chandler 1994; Chandler and Ribeiro 1995; Chandler and Gatt 2000; Chandler and Blasco-Zumeta 2001; Chandler et al. 2006; Banamar et al. 2020, Driauach et al. 2022; El Mouden et al. 2024). However, the distribution of these species over the five countries of North Africa is unbalanced and unequal; the fungus gnat fauna of some countries of the region, for example, Libya, is unknown, and that of Egypt is poorly defined with only two Keroplatidae species from one genus. As for Algeria and Tunisia, the knowledge of this dipteran group is modest as there have been no specific studies of fungus gnats in those countries, with all records having been obtained casually by general recording of Diptera. In Algeria, there are records of 29 species of fungus gnats from 17 genera comprising one species of Bolitophilidae from one genus, five species of Keroplatidae from three genera and 23 species of Mycetophilidae belonging to 13 genera. Meanwhile, the Tunisian fauna gathers 34 fungus gnat species from 23 genera belonging to two families: Keroplatidae, with nine species from seven genera and Mycetophilidae, with 25 species distributed in 16 genera (see Table 2).

Among the North African countries, Morocco has the best-known fungus gnat fauna. While only ten species of Mycetophilidae and two species of Keroplatidae were recorded from Morocco before 2020, the recent studies led by Banamar et al. (2020), Driauach et al. (2022), and El Mouden et al. (2024) improved the knowledge of this group by recording the following species as new to Morocco: 66 species of Mycetophilidae, ten species of Keroplatidae, and one species of the family Bolitophilidae. Overall, the Moroccan fauna was thus increased to 76 species of Mycetophilidae distributed in 28 genera, 12 species of Keroplatidae belonging to seven genera, and one species of Bolitophilidae. The largest share of these records belongs to the Rif area, while only a few records were from other regions of the Moroccan territory. Consequently, this paper presents the first concentrated study of this dipteran group from the Middle Atlas region, one of the most unstudied areas of Morocco. Thus, a total of 69 fungus gnat species (1 species of Bolitophilidae, 3 species of Keroplatidae, and 65 species of Mycetophilidae), of the 71 species mentioned in this study, are recorded for the first time in the Middle Atlas Mountains. Among the fauna identified, the Mycetophilidae genus *Allodia* Winnertz, 1864, is new to Morocco, while the genus *Synplasta* Skuse, 1890 is new to both Morocco and North Africa. Furthermore, out of the 71 studied fungus gnat species, 19 species (1 species of Keroplatidae and 18 species of Mycetophilidae) are recorded for the first time in Morocco, of which 18 are new to North Africa. Therefore, as expected in our previous study (El Mouden et al. 2024), Morocco appears to be home to more endemic fungus gnat species as this paper presents the description of the new species *Docosiatazekkae* sp. nov., which raises the number of endemic species of Mycetophilidae of Morocco to six species in total. *Docosiatazekkae* sp. nov. resembles many other species of Mediterranean *Docosia* Winnertz, 1864, although it is distinguished by some morphological characters detailed in this paper.

## ﻿Material and methods

### ﻿Area of study

The current study is situated in Morocco, one of the “five” countries of North Africa, a geographic entity that has no single accepted definition. It has been regarded by some as stretching from the Atlantic shores of Morocco in the west to the Suez Canal and the Red Sea in the east. Others have limited it to the countries of Morocco, Algeria, and Tunisia, a region known by the French during colonial times as Afrique du Nord and by the Arabs as the Maghrib (“West”). Our study took part in four Moroccan protected areas but mainly in the National Park of Tazekka (**PNTZK**), one of the ten protected Parks of Morocco, located in the northernmost eastern part of the Middle Atlas, near the city of Taza. It was created by Visiriel Decree of July 11, 1950, to protect all the natural resources of Jbel Tazekka summit (culminating at 1,980 metres), and more particularly, the cedar forest (*Cedrusatlantica*), which stands solely on this peak. Since its creation, the Tazekka National Park has expanded from originally 680 hectares (1950) to 13,737 ha (2004) to ensure the protection of more faunal and floral biodiversity by protecting more nearby natural areas and hydrological resources (Elgazzane 2019). The second national park that is part of the Middle Atlas region is the National Park of Ifrane (**PNI**). It is located at the central part of the Middle Atlas Mountains, almost entirely within the Sebou watershed. It was created in 2004 and initially included 53,800 ha. In 2008, the PNI was extended to 125,000 ha including 65,290 ha of forests which is equal to almost all the forests of the province of Ifrane. It contains, thus, 10% of the world surface of the Atlas cedar which classifies it in the heart of the Atlas Cedar Biosphere Reserve (Ismaili Alaoui et al. 2022). In the Rif region, our study included The National Park of Talassemtane (**PNTLS**), which is characterised by a significant biome for faunal and floral diversity in Morocco and North Africa. It is overlooking the Mediterranean Sea and takes part of the Intercontinental Biosphere Reserve of the Mediterranean. The National Park of Talassemtane is biogeographically located in the Rif region western part of northern Morocco, in a landlocked area of the Western Rif chain of the Tangier-Tetouan-Al Hoceima region. It covers an area of 58,022 ha distributed between two provinces of Chefchaouen (78%) and Tétouan (22%) and extends over nine rural communes including six belonging to the province of Chefchaouen (Tassif, Talembote, Bab Taza, Beni Selmane and Bni Derkoul, Tassift) and three to that of Tétouan (El Hamra, Oulad Ali Manson and Al Ouad) (Aoulad-Sidi-Mhend et al. 2020). Not far away from the National Park of Talassemtane, the territory of the Natural Park of Bouhachem (**PPNB**), which takes its name from the Bouhachem mausoleum existing in the area, is located in the northwest of Morocco in the biogeographic region of the Western Rif, precisely on the southern side of the Mediterranean basin. The PPNB is entirely included in the Tangier-Tétouan region, where it extends over six rural communes belonging to the three provinces of Tétouan, Larache and Chefchaouen: Béni Leit and Al Ouad (Province of Tétouan), Derdara, Laghdir and Tanakoub (Province of Chefchaouen) and Tazroute (Province of Larache). It covers an area of 75,497 ha of which 50,113 ha belongs to the province of Chefchaouen, and 40–50% of the total area belongs to the forest domain (Bachar et al. 2021). It constitutes a rural region characterised by a distinct identity and a wealth of natural and cultural heritage. However, this balance remains fragile and increasingly threatened. Consequently, it has been incorporated into heritage preservation initiatives by public authorities.

### ﻿Methodology

**Figure 1.**
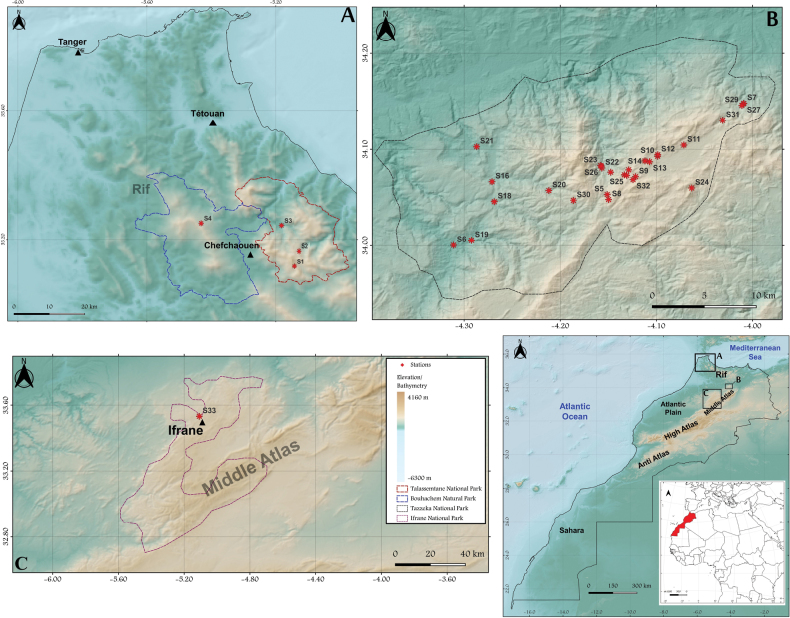
Map of study areas within Morocco **A** the NP of Talassemtane (in red) and the Natural Park of Bouhachem (in blue) **B** the NP of Tazekka **C** the NP of Ifrane: localities within corresponding national parks are indicated by red stars and nearby cities with triangles .

Collecting was performed in 33 sampling sites (Table 1, Fig. 1) of which 28 were distributed over mountainous areas of the National Park of Tazekka, such as forests, rivers, lakes, waterfalls, entrances of caves, and five additional sites distributed between the three other protected Parks (PNTLS, PNI, and PPNB). The sampling results contained a total of 4,216 specimens of Mycetophilidae (3,817 males and 399 females), eight specimens of Keroplatidae, all of them males, and one male of Bolitophilidae; all the specimens were collected using the sweeping technique. The material was collected by members of the Group Belqat, PhD students: I. Saidoun, K. Aattouch, M. Beni-Eich, M. El Ouahabi, and the first author during the period 10 February 2020–2 May 2023 and was preserved in 96% ethanol. The material was sorted by the first author and was identified to generic level with the help of Dr. P. Beuk. Species identifications were done by Dr. P.J. Chandler. The examined material will be deposited in the collection of Diptera of the Department of Biology, Faculty of Sciences, University Abdelmalek Essaadi, Tétouan (**UAE-FST**). The holotype of the newly described species will be deposited at the Natural History Museum, London, UK (**NHMUK**). An annotated list of fungus gnat species reported from Morocco in the present study is provided, in alphabetical order, with the mention of the North African distribution of each species.

**Table 1.** Sampling sites (in alphabetical order) hosting the species collected in the four Moroccan protected areas, with altitudes and geographical coordinates.

Station	Elevation (m)	Geographical coordinates
		Latitude, Longitude
Rif Mountains		
National Park of Talassemtane		
1. Forêt Malâab Tizimezzan	1452	35.1156, -5.1388
2. Oued Bni Mhamed	1314	35.1603, -5.1269
3. Oued Farda	420	35.2392, -5.1743
Natural Park Project of Bouhachem		
4. Forêt après Amsemlil	1127	35.2579, -5.4347
Middle Atlas Mountains		
National Park of Tazekka		
5. Aïn Admam	1453	34.0530, -4.1512
6. Barrage Bab Lota	615	34.0003, -4.3111
7. Cascade Ras Lma	985	34.1480, -4.0090
8. Forêt Admam	1500	34.0479, -4.1497
9. Forêt Bab Boudir	1450	34.0716, -4.1216
10. Forêt Bouhayati	1430	34.0928, -4.0987
11. Forêt gouffre Friouato	1503	34.1045, -4.0714
12. Gouffre Izora	1430	34.0944, -4.0985
13. Grotte Bouhayati	1424	34.0871, -4.1071
14. Grotte Bouslama	1449	34.0879, -4.1118
15. Lacune Bab Boudir	1474	34.0786, -4.1288
16. Maison forestière	806	34.0663, -4.2709
17. Oued Aïn Chabab	1389	34.0735, -4.1333
18. Oued Azhar	801	34.0456, -4.2686
19. Oued Boussbaâ	597	34.0053, -4.2924
20. Oued El Ghannaj	958	34.0571, -4.2118
21. Oued Lagziri	650	34.1026, -4.2870
22. Oued Lek’hal	1053	34.0806, -4.1567
23. Oued Sidi Boulaâla	1097	34.0831, -4.1580
24. Oued Tametrhouste	1413	34.0598, -4.0632
25. Oued Taourirt	1343	34.0729, -4.1308
26. Pont Oued Lek’hal	1053	34.0825, -4.1572
27. Pont Ras Lma	1972	34.1476, -4.0093
28. Ruisseau Aïn Chabab	1261	34.0762, -4.1475
29. Ruisselet Ras Lma	1022	34.1457, -4.0110
30. Vallée des cerfs	1353	34.0469, -4.1863
31. Vallée des oiseaux	1318	34.1302, -4.0310
32. Village Bab Boudir	1579	34.0685, -4.1243
National Park of Ifrane		
33. Forêt Bab Lkhayl	1655.6	33.5320, -5.1081

## ﻿Taxonomy

### ﻿Family Bolitophilidae

**Genus *Bolitophila* Meigen, 1818**

#### ﻿ Bolitophila saundersii

Taxon classificationAnimaliaDipteraBolitophilidae

(Curtis, 1836)

BDBF29CA-92B5-5CAA-B44D-3B35FE8C5A81

##### Material examined.

Morocco – **Middle Atlas Region** • 1♂; Oued Taourirt; 20/V/2022; K. Aattouch, M. El Ouahabi and M.A. El Mouden leg; UAE-FST MA22/2402.

##### North African distribution.

Algeria (Burghele-Balacesco 1967); Morocco (Driauach et al. 2022).

### ﻿Family Keroplatidae

**Subfamily Macrocerinae Rondani, 1856**

**Genus *Macrocera* Meigen, 1804**

#### ﻿ Macrocera fasciata

Taxon classificationAnimaliaDipteraKeroplatidae

Meigen, 1804

E29BA53D-46A5-512A-A947-05C51738CD25

##### Material examined.

Morocco – **Middle Atlas Region** • 1♂; Forêt Admam; 22/V/2022; K. Aattouch, M. El Ouahabi and M.A. El Mouden leg; UAE-FST MA22/2403.

##### North African distribution.

Algeria (Becker 1907); Morocco (Driauach et al. 2022).

### ﻿Subfamily Keroplatinae Rondani, 1856

**Tribe Orfeliini Malloch, 1917**

**Genus *Neoplatyura* Malloch, 1928**

#### ﻿ Neoplatyura biumbrata

Taxon classificationAnimaliaDipteraKeroplatidae

(Edwards, 1913)

3D8C3F87-6A20-5FEE-9A3C-E58196BA224D

##### Material examined.

Morocco – **Middle Atlas Region** • 3♂♂; Grotte Bouslama; 19/05/2022; K. Aattouch, M. El Ouahabi and M.A. El Mouden leg; UAE-FST MA22/2404 • 1♂; Pont Ras Lma; 19/V/2022; K. Aattouch, M. El Ouahabi and M.A. El Mouden leg; UAE-FST MA22/2405.

##### North African distribution.

Morocco (Driauach et al. 2022).

###### ﻿Genus *Orfelia* Costa, 1857

#### ﻿ Orfelia fasciata

Taxon classificationAnimaliaDipteraKeroplatidae

(Meigen, 1804)

C3003182-30F4-524B-8A88-0D5B3C790EE7

##### Material examined.

Morocco – **Middle Atlas Region** • 1♂; Cascade Ras Lma; 19/V/2022; K. Aattouch, M. El Ouahabi and M.A. El Mouden leg; UAE-FST MA22/2406 • 1♂; Oued Lek’hal; 20/V/2022; K. Aattouch, M. El Ouahabi and M.A. El Mouden leg; UAE-FST MA22/2407 • 1♂; Oued El Ghannaj; 22/V/2022; K. Aattouch, M. El Ouahabi and M.A. El Mouden leg; UAE-FST MA22/2408.

##### North African distribution.

New to Morocco and North Africa.

### ﻿Family Mycetophilidae

**Subfamily Gnoristinae Edwards,1925**

**Genus *Boletina* Staeger, 1840**

#### ﻿ Boletina lundstroemi

Taxon classificationAnimaliaDipteraMycetophilidae

Landrock, 1912

795FF888-9399-56D7-AED3-406712B1E441

##### Material examined.

Morocco – **Middle Atlas Region** • 1♂; Oued Aïn Chabab; 20/V/2022; K. Aattouch, M. El Ouahabi and M.A. El Mouden leg; UAE-FST MA22/2409.

##### North African distribution.

New to Morocco and North Africa.

###### ﻿Genus *Coelosia* Winnertz, 1864

#### ﻿ Coelosia fusca

Taxon classificationAnimaliaDipteraMycetophilidae

Bezzi, 1892

36315DA8-7FD5-544E-A276-FF981FD4B15A

##### Material examined.

Morocco – **Middle Atlas Region** • 1♂, 2♀♀; Gouffre Izora; 19/V/2022; K. Aattouch, M. El Ouahabi and M.A. El Mouden leg; UAE-FST MA22/2410.

##### North African distribution.

Morocco (Banamar et al. 2020).

###### ﻿Genus *Docosia* Winnertz, 1864

#### ﻿ Docosia gilvipes

Taxon classificationAnimaliaDipteraMycetophilidae

(Walker, 1856)

62ECF684-B27B-5430-AEFB-21962641DF8B

##### Material examined.

Morocco – **Middle Atlas Region** • 6♂♂; Gouffre Izora; 19/V/2022; K. Aattouch, M. El Ouahabi and M.A. El Mouden leg; UAE-FST MA22/2411 • 1♀; Grotte Bouslama; 19/V/2022; K. Aattouch, M. El Ouahabi and M.A. El Mouden leg; UAE-FST MA22/2412 • 1♂; Vallée des oiseaux; 19/V/2022; K. Aattouch, M. El Ouahabi and M.A. El Mouden leg; UAE-FST MA22/2413 • 6♂♂; Grotte Bouhayati; 20/V/2022; K. Aattouch, M. El Ouahabi and M.A. El Mouden leg; UAE-FST MA22/2414.

##### North African distribution.

Morocco (Banamar et al. 2020).

#### ﻿ Docosia melita

Taxon classificationAnimaliaDipteraMycetophilidae

Chandler & Gatt, 2000

CF33AC7E-4023-54B4-B0C0-FF91A2D35A48

##### Material examined.

Morocco – **Middle Atlas Region** • 3♂♂; Vallée des oiseaux; 19/V/2022; K. Aattouch, M. El Ouahabi and M.A. El Mouden leg; UAE-FST MA22/2415 • 1♂; Grotte Bouhayati; 20/V/2022; K. Aattouch, M. El Ouahabi and M.A. El Mouden leg; UAE-FST MA22/2416 • 2♂♂; Oued Sidi Boulaâla; 20/V/2022; K. Aattouch, M. El Ouahabi and M.A. El Mouden leg; UAE-FST MA22/2417 • 5♂♂; Oued Taourirt; 20/V/2022; K. Aattouch, M. El Ouahabi and M.A. El Mouden leg; UAE-FST MA22/2418 • 1♂; Ruisseau Aïn Chabab; 20/V/2022; K. Aattouch, M. El Ouahabi and M.A. El Mouden leg; UAE-FST MA22/2419 • 1♂; Aïn Admam; 22/V/2022; K. Aattouch, M. El Ouahabi and M.A. El Mouden leg; UAE-FST MA22/2420 • 1♂; Oued Lgziri; 23/V/2022; K. Aattouch, M. El Ouahabi and M.A. El Mouden leg; UAE-FST MA22/2421.

##### North African distribution.

Morocco (El Mouden et al. 2024).

#### ﻿ Docosia tazekkae

Taxon classificationAnimaliaDipteraMycetophilidae

Chandler, El Mouden & Belqat sp. nov.

F146DC2C-36DC-55BB-9798-CE745AAAC234

https://zoobank.org/A58CAAC4-8605-4E8B-BA05-860D8DF141E5

##### Type material.

***Holotype*.** Morocco – **Middle Atlas Region** • ♂ (mounted in DMHF, terminalia on a slide); Forêt Bab Boudir; 20/V/2022; K. Aattouch, M. El Ouahabi and M.A. El Mouden leg; collected using sweep net; NHMUK. ***Paratypes*.** Morocco – **Middle Atlas Region** • 1♂; Forêt Bab Lkhayl; 2/V/2023; M. Beni-Eich, K. Aattouch and M.A. El Mouden leg; collected using sweep net; UAE-FST MA23/2401. – **Rif Region** • 1♂; Forêt après Amsemlil; 10/II/2020; Group Belqat leg; collected using sweep net; UAE-FST R22/2450 • 1♂; Forêt Malâab Tizimezzan; 12/V/2022; M. Beni-Eich, K. Aattouch, M. El Ouahabi and M.A. El Mouden leg; collected using sweep net; UAE-FST R22/2451 • 2♂♂ (mounted in DMHF, terminalia of one Oued Bni Mhamed specimen on a slide); Oued Bni Mhamed; 11/V/2022; M. Beni-Eich, K. Aattouch, M. El Ouahabi and M.A. El Mouden leg; collected using sweep net; UAE-FST R22/2449 • 1♂; Oued Farda; 11/III/2023; I. Saidoun leg; collected using sweep net; UAE-FST R22/2452.

##### Diagnosis.

This species resembles several other Mediterranean species of this genus, including *D.melita* Chandler & Gatt, 2000 previously recorded from Morocco (El Mouden et al. 2024) in its dark body colouration, mainly yellow legs and bare laterotergites. It belongs to a group of species with a simple gonostylus bearing a row of spines on its inner margin. Within this group it is most similar to *D.kerkini* Kurina & Ševčík, 2011, described from Greece (Kurina and Ševčík 2011) which also has a medioventral humped process of the apical margin of the gonocoxites bearing a cluster of short spinose setae on each side preapically. In *D.kerkini* the row of the spines on the gonostylus is longer (12 spines compared to 8), and the medioventral process of the gonocoxites is shorter and narrower with a deeper medial excavation and more tight patches of stout setae; tergite 9 is also differently shaped, broadened apically with a concave apical margin.

##### Description.

**Figure 2.**
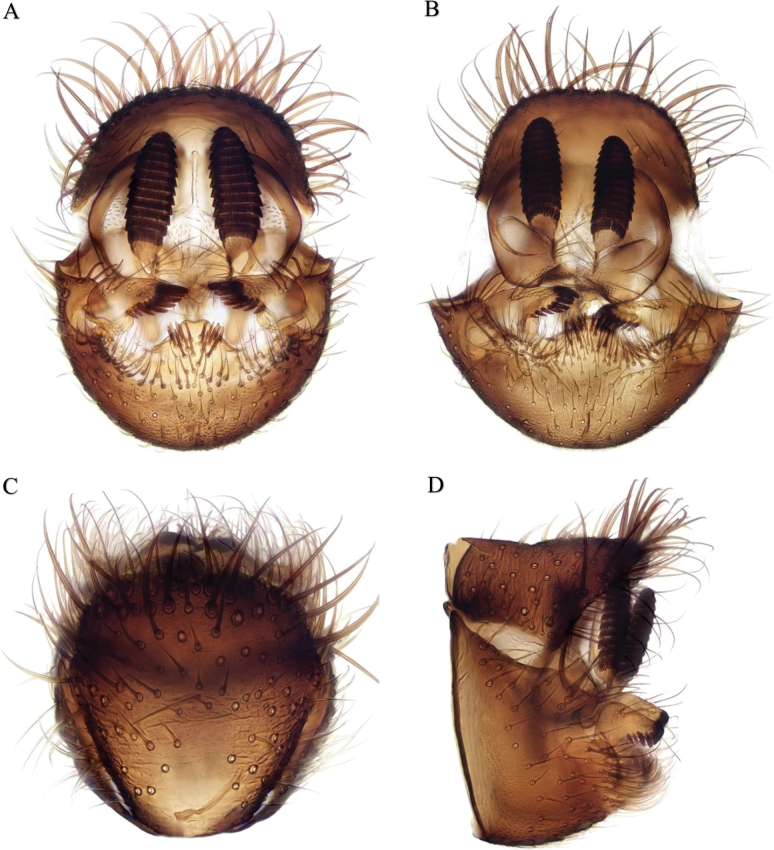
*D.tazekkae* Chandler, El Mouden & Belqat, sp. nov. Male terminalia posterior view (tergite dorsally, apical margin of gonocoxites ventrally) of **A** holotype and same of **B** paratype from Oued Bni Mhamed. Holotype terminalia in **C** dorsal and **D** lateral views.

**Male.** Wing length 3.0–3.2 mm. ***Colouration*.** Head including antennae and body including terminalia all black. Legs mostly yellow. Coxae dark on basal part (extreme base of fore and mid coxa, ~ 1/3–1/2 of hind coxa); trochanters dark, hind femur narrowly dark at base and tip. Wing clear, with costa and radial veins dark, other veins pale. Haltere yellow. ***Head*.** Antenna longer than head and thorax together, with slender flagellomeres > 2 × as long as broad. Ocelli situated close to eye margin. ***Thorax*.** Laterotergite bare. ***Legs*.** Mid tibia with four short anterior and three or four longer dorsal setae, almost as long as tibial width. Hind tibia with 10–12 short anterior and six longer dorsal setae, about as long as tibial width, with a few short setae in line beyond them. ***Wing*.** Sc ends in R ~ 1/2 or a little more to base of Rs. R_1_ 2–2.5 × length of r-m. ***Terminalia*** (Fig. 2). Tergite 9 short and broad, rounded apically with long setae on apical margin. Cercus with tiers of close-set combs of retinacula (12 combs in holotype, 13 in paratype figured). Gonostylus simple, narrowed apically, with long fine scattered setae on outer surface and a row of eight strong spinose setae on inner margin, the most distal longest, the proximal two or three shorter than the rest. Gonocoxites with a medioventral humped process of the apical margin, bearing an irregular cluster of short spinose setae on each side preapically, the bare part beyond these spines with a shallow apical emargination; the apical margin on each side bearing a row of short strong setae and a narrow internal flange bearing long fine setae.

**Female.** Unknown.

##### Etymology.

**Figure 3.**
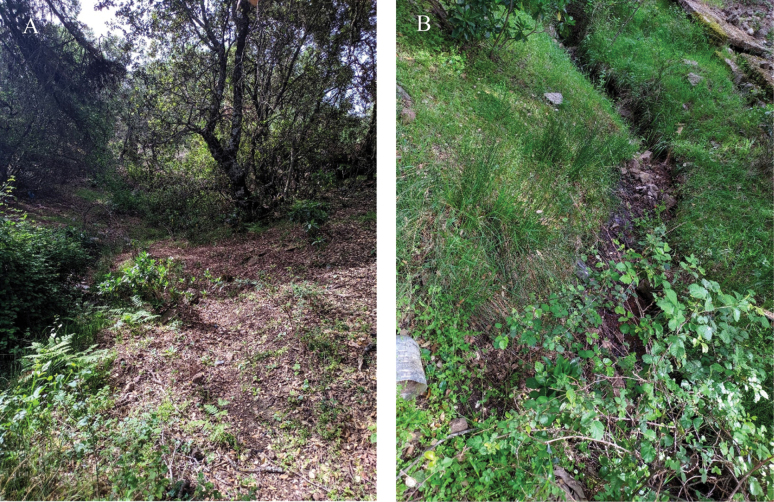
Moroccan habitat of *Docosiatazekkae* Chandler, El Mouden & Belqat, sp. nov. Environments of the Forêt Bab Boudir within the PNTZK**A** Bab Boudir forest **B** Bab Boudir rivulet.

After the name of the Tazekka National Park, which shelters the new species in the Bab Boudir Forest locality (Fig. 3).

##### Habitat.

The holotype of this species was collected in the remains of a forest that neighbours the holiday centre of Bab Boudir, in the heart of the Tazekka National Park (Fig. 3). The sampling took place at ca 0800 hrs on a sunny morning (25 °C), with low humidity (17%) and moderate wind speed (14 Km/h) in a dense part of the forest where the tree branches seem to meet as they are very close to each other, over a very small river (Bab Boudir rivulet), with calming low speed waters. The dense vegetative cover creates a shadowy, sheltered and fairly cold spot where sun rays can barely penetrate.

###### ﻿Genus *Megophthalmidia* Dziedzicki, 1889

#### ﻿ Megophthalmidia amsemlili

Taxon classificationAnimaliaDipteraMycetophilidae

Chandler, Belqat & Banamar, 2024

2331B156-76F0-5705-80D8-F940034290EC

##### Literature records.

Morocco – **Middle Atlas Region** • 1♂; Oued Taourirt; 20/V/2022; K. Aattouch, M. El Ouahabi and M.A. El Mouden leg; UAE-FST MA22/2401 (El Mouden et al. 2024).

##### North African distribution.

Morocco (El Mouden et al. 2024).

###### ﻿Genus *Synapha* Meigen, 1818

#### ﻿ Synapha fasciata

Taxon classificationAnimaliaDipteraMycetophilidae

Meigen, 1818

4B4278B1-27A6-55AA-871E-DE31D874F5FD

##### Material examined.

Morocco – **Middle Atlas Region** • 1♂; Forêt Admam; 22/V/2022; K. Aattouch, M. El Ouahabi and M.A. El Mouden leg; UAE-FST MA22/2423.

##### North African distribution.

Morocco (Banamar et al. 2020).

###### ﻿Genus *Tetragoneura* Winnertz, 1846

#### ﻿ Tetragoneura

Taxon classificationAnimaliaDipteraMycetophilidae

sp.

8ADB0797-7E34-55BA-A6B3-7F7EA49D15EA

##### Material examined.

Morocco – **Middle Atlas Region** • 1♀; Forêt Admam; 22/V/2022; K. Aattouch, M. El Ouahabi and M.A. El Mouden leg; UAE-FST MA22/2424.

### ﻿Subfamily Leiinae Edwards, 1925

**Genus *Leia* Meigen, 1818**

#### ﻿ Leia arcana

Taxon classificationAnimaliaDipteraMycetophilidae

Chandler, Belqat & Driauach, 2024

C00001E1-DF29-5BFB-B9FE-B626B6CEDB98

##### Material examined.

Morocco – **Middle Atlas Region** • 1♂; Forêt gouffre Friouato; 19/V/2022; K. Aattouch, M. El Ouahabi and M.A. El Mouden leg; UAE-FST MA22/2426 • 7♂♂; Gouffre Izora; 19/V/2022; K. Aattouch, M. El Ouahabi and M.A. El Mouden leg; UAE-FST MA22/2427 • 31♂♂; Grotte Bouslama; 19/V/2022; K. Aattouch, M. El Ouahabi and M.A. El Mouden leg; UAE-FST MA22/2428 • 1♂; Ruisselet Ras Lma; 19/V/2022; K. Aattouch, M. El Ouahabi and M.A. El Mouden leg; UAE-FST MA22/2429 • 4♂♂; Vallée des oiseaux; 19/V/2022; K. Aattouch, M. El Ouahabi and M.A. El Mouden leg; UAE-FST MA22/2430 • 1♂; Village Bab Boudir; 19/V/2022; K. Aattouch, M. El Ouahabi and M.A. El Mouden leg; UAE-FST MA22/2431 • 3♂♂; Forêt Bab Boudir; 20/V/2022; K. Aattouch, M. El Ouahabi and M.A. El Mouden leg; UAE-FST MA22/2425 • 3♂♂; Grotte Bouhayati; 20/V/2022; K. Aattouch, M. El Ouahabi and M.A. El Mouden leg; UAE-FST MA22/2432 • 10♂♂; Oued Aïn Chabab; 20/V/2022; K. Aattouch, M. El Ouahabi and M.A. El Mouden leg; UAE-FST MA22/2433 • 9♂♂; Oued Taourirt; 20/V/2022; K. Aattouch, M. El Ouahabi and M.A. El Mouden leg; UAE-FST MA22/2434 • 1♂; Aïn Admam; 22/V/2022; K. Aattouch, M. El Ouahabi and M.A. El Mouden leg; UAE-FST MA22/2435 • 3♂♂; Forêt Admam; 22/V/2022; K. Aattouch, M. El Ouahabi and M.A. El Mouden leg; UAE-FST MA22/2436.

##### North African distribution.

Morocco (El Mouden et al. 2024).

#### ﻿ Leia beckeri

Taxon classificationAnimaliaDipteraMycetophilidae

Landrock, 1940

54686A91-9797-55E9-A63C-54FC795B800C

##### Material examined.

Morocco – **Middle Atlas Region** • 1♀; Oued Boussbaâ; 22/V/2022; K. Aattouch, M. El Ouahabi and M.A. El Mouden leg; UAE-FST MA22/2437.

##### North African distribution.

Algeria (Becker 1907 as *L.bifasciata*; Hackman et al. 1988; Chandler 2009); Morocco (Banamar et al. 2020).

#### ﻿ Leia bimaculata

Taxon classificationAnimaliaDipteraMycetophilidae

(Meigen, 1804)

3FA0FFCA-C61A-5F36-B823-3D3B41D1A50A

##### Material examined.

Morocco – **Middle Atlas Region** • 1♂; Forêt Bouhayati; 19/V/2022; K. Aattouch, M. El Ouahabi and M.A. El Mouden leg; UAE-FST MA22/2438 • 2♂♂; Forêt gouffre Friouato; 19/V/2022; K. Aattouch, M. El Ouahabi and M.A. El Mouden leg; UAE-FST MA22/2439 • 1♂; Gouffre Izora; 19/V/2022; K. Aattouch, M. El Ouahabi and M.A. El Mouden leg; UAE-FST MA22/2440 • 1♂; Grotte Bouslama; 19/V/2022; K. Aattouch, M. El Ouahabi and M.A. El Mouden leg; UAE-FST MA22/2441 • 1♂; Vallée des oiseaux; 19/V/2022; K. Aattouch, M. El Ouahabi and M.A. El Mouden leg; UAE-FST MA22/2442 • 3♂♂; Grotte Bouhayati; 20/V/2022; K. Aattouch, M. El Ouahabi and M.A. El Mouden leg; UAE-FST MA22/2443 • 1♂; Pont Oued Lek’hal; 20/V/2022; K. Aattouch, M. El Ouahabi and M.A. El Mouden leg; UAE-FST MA22/2444 • 1♂; Ruisseau Aïn Chabab; 20/V/2022; K. Aattouch, M. El Ouahabi and M.A. El Mouden leg; UAE-FST MA22/2445 • 1♂; Oued Tametrhouste; 21/V/2022; K. Aattouch, M. El Ouahabi and M.A. El Mouden leg; UAE-FST MA22/2446 • 1♂; Forêt Admam; 22/V/2022; K. Aattouch, M. El Ouahabi and M.A. El Mouden leg; UAE-FST MA22/2447.

##### North African distribution.

Algeria (Burghele-Balacesco 1972); Morocco (Chandler et al. 2006; Banamar et al. 2020).

###### ﻿Genus *Novakia* Strobl, 1893

#### ﻿ Novakia scatopsiformis

Taxon classificationAnimaliaDipteraMycetophilidae

Strobl, 1893

0A9B1A5A-5337-5B97-99EA-EDEDBBF2C151

##### Material examined.

Morocco – **Middle Atlas Region** • 6♂♂; Gouffre Izora; 19/V/2022; K. Aattouch, M. El Ouahabi and M.A. El Mouden leg; UAE-FST MA22/2448 • 3♂♂; Grotte Bouslama; 19/V/2022; K. Aattouch, M. El Ouahabi and M.A. El Mouden leg; UAE-FST MA22/2449 • 2♂♂; Vallé des oiseaux; 19/V/2022; K. Aattouch, M. El Ouahabi and M.A. El Mouden leg; UAE-FST MA22/2450 • 13♂♂; Oued Taourirt; 20/V/2022; K. Aattouch, M. El Ouahabi and M.A. El Mouden leg; UAE-FST MA22/2451 • 2♀♀; Barrage Bab Lota; 22/V/2022; K. Aattouch, M. El Ouahabi and M.A. El Mouden leg; UAE-FST MA22/2452.

##### North African distribution.

Tunisia (Enderlein 1913; Hackman et al. 1988; Chandler 1994); Morocco (Banamar et al. 2020).

#### ﻿ Novakia simillima

Taxon classificationAnimaliaDipteraMycetophilidae

Strobl, 1910

03533AD7-251B-5A80-8579-6689E6DD8481

##### Material examined.

Morocco – **Middle Atlas Region** • 1♂; Grotte Bouslama; 19/V/2022; K. Aattouch, M. El Ouahabi and M.A. El Mouden leg; UAE-FST MA22/2453.

##### North African distribution.

Morocco (Banamar et al. 2020).

### ﻿Subfamily Mycetophilinae Newman, 1834

**Tribe Exechiini Edwards, 1925**

**Genus *Allodia* Winnertz, 1864**

#### ﻿ Allodia ornaticollis

Taxon classificationAnimaliaDipteraMycetophilidae

(Meigen, 1818)

31A717C8-5EE5-529B-A2CA-6A8864AFFBF1

##### Material examined.

Morocco – **Middle Atlas Region** • 1♂; Gouffre Izora; 19/V/2022; K. Aattouch, M. El Ouahabi and M.A. El Mouden leg; UAE-FST MA22/2454.

##### North African distribution.

New to Morocco and North Africa.

###### ﻿Genus *Brevicornu* Marshall, 1896

#### ﻿ Brevicornu fissicauda

Taxon classificationAnimaliaDipteraMycetophilidae

(Lundström, 1911)

A633525A-807A-54D0-850C-1C0F470ACAFB

##### Material examined.

Morocco – **Middle Atlas Region** • 1♂; Oued Taourirt; 20/V/2022; K. Aattouch, M. El Ouahabi and M.A. El Mouden leg; UAE-FST MA22/2455.

##### North African distribution.

New to Morocco and North Africa.

#### ﻿ Brevicornu griseicolle

Taxon classificationAnimaliaDipteraMycetophilidae

Staeger, 1840

96E83FB4-36F8-5DCB-AF09-36EA443185A8

##### Material examined.

Morocco – **Middle Atlas Region** • 740♂♂; Gouffre Izora; 19/V/2022; K. Aattouch, M. El Ouahabi and M.A. El Mouden leg; UAE-FST MA22/2457 • 161♂♂; Grotte Bouslama; 19/V/2022; K. Aattouch, M. El Ouahabi and M.A. El Mouden leg; UAE-FST MA22/2458 • 1♂; Pont Ras Lma; 19/V/2022; K. Aattouch, M. El Ouahabi and M.A. El Mouden leg; UAE-FST MA22/2459 • 6♂♂; Vallée des oiseaux; 19/V/2022; K. Aattouch, M. El Ouahabi and M.A. El Mouden leg; UAE-FST MA22/2460 • 1♂; Forêt Bab Boudir; 20/V/2022; K. Aattouch, M. El Ouahabi and M.A. El Mouden leg; UAE-FST MA22/2456 • 26♂♂; Grotte Bouhayati; 20/V/2022; K. Aattouch, M. El Ouahabi and M.A. El Mouden leg; UAE-FST MA22/2461 • 2♂♂; Oued Aïn Chabab; 20/V/2022; K. Aattouch, M. El Ouahabi and M.A. El Mouden leg; UAE-FST MA22/2462 • 2♂♂; Oued Taourirt; 20/V/2022; K. Aattouch, M. El Ouahabi and M.A. El Mouden leg; UAE-FST MA22/2463 • 1♂; Pont Oued Lek’hal; 20/V/2022; K. Aattouch, M. El Ouahabi and M.A. El Mouden leg; UAE-FST MA22/2464 • 2♂♂; Aïn Admam; 22/V/2022; K. Aattouch, M. El Ouahabi and M.A. El Mouden leg; UAE-FST MA22/2465 • 5♂♂; Forêt Admam; 22/V/2022; K. Aattouch, M. El Ouahabi and M.A. El Mouden leg; UAE-FST MA22/2466.

##### North African distribution.

Morocco (Banamar et al. 2020).

#### ﻿ Brevicornu intermedium

Taxon classificationAnimaliaDipteraMycetophilidae

(Santos Abréu, 1920)

77EF9A8D-1B01-5A75-A2FA-148E71E1DF92

##### Material examined.

Morocco – **Middle Atlas Region** • 214♂♂; Gouffre Izora; 19/V/2022; K. Aattouch, M. El Ouahabi and M.A. El Mouden leg; UAE-FST MA22/2467 • 134♂♂; Grotte Bouslama; 19/V/2022; K. Aattouch, M. El Ouahabi and M.A. El Mouden leg; UAE-FST MA22/2468 • 1♂; Vallée des oiseaux; 19/V/2022; K. Aattouch, M. El Ouahabi and M.A. El Mouden leg; UAE-FST MA22/2469 • 29♂♂; Grotte Bouhayati; 20/V/2022; K. Aattouch, M. El Ouahabi and M.A. El Mouden leg; UAE-FST MA22/2470 • 1♂; Oued Sidi Boulaâla; 20/V/2022; K. Aattouch, M. El Ouahabi and M.A. El Mouden leg; UAE-FST MA22/2471 • 11♂♂; Aïn Admam; 22/V/2022; K. Aattouch, M. El Ouahabi and M.A. El Mouden leg; UAE-FST MA22/2472 • 1♂; Forêt Admam; 22/V/2022; K. Aattouch, M. El Ouahabi and M.A. El Mouden leg; UAE-FST MA22/2473.

##### North African distribution.

Morocco (Banamar et al. 2020).

#### ﻿ Brevicornu sericoma

Taxon classificationAnimaliaDipteraMycetophilidae

(Meigen, 1830)

9102F52E-C07F-58C5-BFCE-048B74379232

##### Material examined.

Morocco – **Middle Atlas Region** • 337♂♂; Gouffre Izora; 19/V/2022; K. Aattouch, M. El Ouahabi and M.A. El Mouden leg; UAE-FST MA22/2474 • 136♂♂; Grotte Bouslama; 19/V/2022; K. Aattouch, M. El Ouahabi and M.A. El Mouden leg; UAE-FST MA22/2475 • 4♂♂; Grotte Bouhayati; 20/V/2022; K. Aattouch, M. El Ouahabi and M.A. El Mouden leg; UAE-FST MA22/2476 • 1♂; Lacune Bab Boudir; 20/V/2022; K. Aattouch, M. El Ouahabi and M.A. El Mouden leg; UAE-FST MA22/2477 • 4♂♂; Oued Aïn Chabab; 20/V/2022; K. Aattouch, M. El Ouahabi and M.A. El Mouden leg; UAE-FST MA22/2478 • 1♂; Pont Oued Lek’hal; 20/V/2022; K. Aattouch, M. El Ouahabi and M.A. El Mouden leg; UAE-FST MA22/2479 • 1♂; Ruisseau Aïn Chabab; 20/V/2022; K. Aattouch, M. El Ouahabi and M.A. El Mouden leg; UAE-FST MA22/2480 • 19♂♂; Forêt Admam; 22/V/2022; K. Aattouch, M. El Ouahabi and M.A. El Mouden leg; UAE-FST MA22/2481 • 5♂♂; Oued Azhar; 22/V/2022; K. Aattouch, M. El Ouahabi and M.A. El Mouden leg; UAE-FST MA22/2482 • 1♂; Oued El Ghannaj; 22/V/2022; K. Aattouch, M. El Ouahabi and M.A. El Mouden leg; UAE-FST MA22/2483.

##### North African distribution.

Tunisia (Chandler et al. 2006); Morocco (Banamar et al. 2020).

#### ﻿ Brevicornu verralli

Taxon classificationAnimaliaDipteraMycetophilidae

(Edwards, 1925)

D26150EE-BCF1-5BA3-B844-DDAA9239475F

##### Material examined.

Morocco – **Middle Atlas Region** • 17♂♂; Gouffre Izora; 19/V/2022; K. Aattouch, M. El Ouahabi and M.A. El Mouden leg; UAE-FST MA22/2485 • 13♂♂; Grotte Bouslama; 19/V/2022; K. Aattouch, M. El Ouahabi and M.A. El Mouden leg; UAE-FST MA22/2486 • 3♂♂; Forêt Bab Boudir; 20/V/2022; K. Aattouch, M. El Ouahabi and M.A. El Mouden leg; UAE-FST MA22/2484 • 1♂; Grotte Bouhayati; 20/V/2022; K. Aattouch, M. El Ouahabi and M.A. El Mouden leg; UAE-FST MA22/2487.

##### North African distribution.

Tunisia (Chandler 1994) and Morocco (Banamar et al. 2020).

###### ﻿Genus *Cordyla* Meigen, 1803

#### ﻿ Cordyla crassicornis

Taxon classificationAnimaliaDipteraMycetophilidae

Meigen, 1818

05778451-3830-5532-9307-22D6A2F19B77

##### Material examined.

Morocco – **Middle Atlas Region** • 1♂; Gouffre Izora; 19/V/2022; K. Aattouch, M. El Ouahabi and M.A. El Mouden leg; UAE-FST MA22/2489 • 2♂♂; Grotte Bouslama; 19/V/2022; K. Aattouch, M. El Ouahabi and M.A. El Mouden leg; UAE-FST MA22/2490 • 1♂; Vallée des oiseaux; 19/V/2022; K. Aattouch, M. El Ouahabi and M.A. El Mouden leg; UAE-FST MA22/2491 • 1♂; Forêt Bab Boudir; 20/V/2022; K. Aattouch, M. El Ouahabi and M.A. El Mouden leg; UAE-FST MA22/2488 • 1♂; Oued Tametrhouste; 21/V/2022; K. Aattouch, M. El Ouahabi and M.A. El Mouden leg; UAE-FST MA22/2492 • 2♂♂; Forêt Admam; 22/V/2022; K. Aattouch, M. El Ouahabi and M.A. El Mouden leg; UAE-FST MA22/2493 • 1♂; Oued Azhar; 22/V/2022; K. Aattouch, M. El Ouahabi and M.A. El Mouden leg; UAE-FST MA22/2494 • 1♂; Maison forestière; 23/V/2022; K. Aattouch, M. El Ouahabi and M.A. El Mouden leg; UAE-FST MA22/2495.

##### North African distribution.

Morocco (Banamar et al. 2020).

#### ﻿ Cordyla murina

Taxon classificationAnimaliaDipteraMycetophilidae

Winnertz, 1864

128069D7-BD89-55D8-B416-DF596709601F

##### Material examined.

Morocco – **Middle Atlas Region** • 1♂; Forêt gouffre Friouato; 19/V/2022; K. Aattouch, M. El Ouahabi and M.A. El Mouden leg; UAE-FST MA22/2496 • 1♂; Grotte Bouslama; 19/V/2022; K. Aattouch, M. El Ouahabi and M.A. El Mouden leg; UAE-FST MA22/2497 • 2♂♂; Oued Aïn Chabab; 20/V/2022; K. Aattouch, M. El Ouahabi and M.A. El Mouden leg; UAE-FST MA22/2498 • 1♂; Oued Taourirt; 20/V/2022; K. Aattouch, M. El Ouahabi and M.A. El Mouden leg; UAE-FST MA22/2499 • 1♂; Vallée des cerfs; 22/V/2022; K. Aattouch, M. El Ouahabi and M.A. El Mouden leg; UAE-FST MA22/24100.

##### North African distribution.

Morocco (Banamar et al. 2020).

###### ﻿Genus *Exechia* Winnertz, 1864

#### ﻿ Exechia dorsalis

Taxon classificationAnimaliaDipteraMycetophilidae

(Staeger, 1840)

5AC77F17-C0F8-5602-A1E9-1F9AF5D11FC0

##### Material examined.

Morocco – **Middle Atlas Region** • 2♂♂; Gouffre Izora; 19/V/2022; K. Aattouch, M. El Ouahabi and M.A. El Mouden leg; UAE-FST MA22/24101 • 1♂; Grotte Bouslama; 19/V/2022; K. Aattouch, M. El Ouahabi and M.A. El Mouden leg; UAE-FST MA22/24102 • 1♂; Grotte Bouhayati; 20/V/2022; K. Aattouch, M. El Ouahabi and M.A. El Mouden leg; UAE-FST MA22/24103 • 3♂♂; Forêt Admam; 22/V/2022; K. Aattouch, M. El Ouahabi and M.A. El Mouden leg; UAE-FST MA22/24104.

##### North African distribution.

Morocco (Banamar et al. 2020).

#### ﻿ Exechia fulva

Taxon classificationAnimaliaDipteraMycetophilidae

Santos Abreu, 1920

1F9174AF-92DA-5E80-9C2A-A8A8F2D269BB

= Rymosiaexornata Séguy, 1941.

##### Material examined.

Morocco – **Middle Atlas Region** • 191♂♂; Gouffre Izora; 19/V/2022; K. Aattouch, M. El Ouahabi and M.A. El Mouden leg; UAE-FST MA22/24106 • 555♂♂; Grotte Bouslama; 19/V/2022; K. Aattouch, M. El Ouahabi and M.A. El Mouden leg; UAE-FST MA22/24107 • 1♂; Forêt Bab Boudir; 20/V/2022; K. Aattouch, M. El Ouahabi and M.A. El Mouden leg; UAE-FST MA22/24105 • 19♂♂; Grotte Bouhayati; 20/V/2022; K. Aattouch, M. El Ouahabi and M.A. El Mouden leg; UAE-FST MA22/24108 • 1♂; Oued Aïn Chabab; 20/V/2022; K. Aattouch, M. El Ouahabi and M.A. El Mouden leg; UAE-FST MA22/24109 • 3♂♂; Aïn Admam; 22/V/2022; K. Aattouch, M. El Ouahabi and M.A. El Mouden leg; UAE-FST MA22/24110.

##### North African distribution.

Tunisia (Chandler and Ribeiro 1995); Morocco (Séguy 1941; Chandler and Ribeiro 1995; Banamar et al. 2020).

#### ﻿ Exechia fusca

Taxon classificationAnimaliaDipteraMycetophilidae

(Meigen, 1804)

28EDA3EC-0B81-5E3D-BFF0-6534554E84CD

##### Material examined.

Morocco – **Middle Atlas Region** • 257♂♂; Gouffre Izora; 19/V/2022; K. Aattouch, M. El Ouahabi and M.A. El Mouden leg; UAE-FST MA22/24111 • 119♂♂; Grotte Bouslama; 19/V/2022; K. Aattouch, M. El Ouahabi and M.A. El Mouden leg; UAE-FST MA22/24112 • 3♂♂; Grotte Bouhayati; 20/V/2022; K. Aattouch, M. El Ouahabi and M.A. El Mouden leg; UAE-FST MA22/24113 • 2♂♂; Aïn Admam; 22/V/2022; K. Aattouch, M. El Ouahabi and M.A. El Mouden leg; UAE-FST MA22/24114.

##### North African distribution.

Algeria (Chandler and Ribeiro 1995); Tunisia (Chandler 1994; Chandler and Ribeiro 1995); Morocco (Banamar et al. 2020).

#### ﻿ Exechia spinuligera

Taxon classificationAnimaliaDipteraMycetophilidae

Lundström, 1912

5E541315-8D7D-545F-8A24-DEDB7B26FB17

##### Material examined.

Morocco – **Middle Atlas Region** • 3♂♂; Gouffre Izora; 19/V/2022; K. Aattouch, M. El Ouahabi and M.A. El Mouden leg; UAE-FST MA22/24115 • 2♂♂; Grotte Bouslama; 19/V/2022; K. Aattouch, M. El Ouahabi and M.A. El Mouden leg; UAE-FST MA22/24116.

##### North African distribution.

New to Morocco and North Africa.

#### ﻿ Exechia neorepanda

Taxon classificationAnimaliaDipteraMycetophilidae

Lindemann, 2021

2129A3FB-0765-5E5E-852C-935F242EFEC0

##### Material examined.

Morocco – **Middle Atlas Region** • 1♂; Grotte Bouslama; 19/V/2022; K. Aattouch, M. El Ouahabi and M.A. El Mouden leg; UAE-FST MA22/24117.

##### North African distribution.

New to Morocco and North Africa.

###### ﻿Genus *Pseudexechia* Tuomikoski, 1966

#### ﻿ Pseudexechia trivittata

Taxon classificationAnimaliaDipteraMycetophilidae

(Stæger, 1840)

9863D395-6B53-5727-9CAA-F3A1F464200A

##### Material examined.

Morocco – **Middle Atlas Region** • 3♂♂; Gouffre Izora; 19/V/2022; K. Aattouch, M. El Ouahabi and M.A. El Mouden leg; UAE-FST MA22/24118 • 2♂♂; Grotte Bouslama; 19/V/2022; K. Aattouch, M. El Ouahabi and M.A. El Mouden leg; UAE-FST MA22/24119.

##### North African distribution.

New to Morocco and North Africa.

#### ﻿ Pseudexechia tuomikoskii

Taxon classificationAnimaliaDipteraMycetophilidae

(Kjærandsen, 2009)

9338DF9E-25CE-5801-9002-40A73394F666

##### Material examined.

Morocco – **Middle Atlas Region** • 10♂♂; Gouffre Izora; 19/V/2022; K. Aattouch, M. El Ouahabi and M.A. El Mouden leg; UAE-FST MA22/24120 • 7♂♂; Grotte Bouslama; 19/V/2022; K. Aattouch, M. El Ouahabi and M.A. El Mouden leg; UAE-FST MA22/24121.

##### North African distribution.

Morocco (Banamar et al. 2020).

###### ﻿Genus *Rymosia* Winnertz, 1864

#### ﻿ Rymosia beaucournui

Taxon classificationAnimaliaDipteraMycetophilidae

Matile, 1963

AC2292F3-47E9-59F9-A109-4E1708BB70DB

##### Material examined.

Morocco – **Middle Atlas Region** • 1♂; Gouffre Izora; 19/V/2022; K. Aattouch, M. El Ouahabi and M.A. El Mouden leg; UAE-FST MA22/24122.

##### North African distribution.

Tunisia (Chandler 1994); Morocco (Chandler and Ribeiro 1995; Chandler et al. 2006; Banamar et al. 2020).

#### ﻿ Rymosia ebejeri

Taxon classificationAnimaliaDipteraMycetophilidae

Chandler & Belqat, 2024

BC3A8859-87C8-5B9B-9C26-16785B6A91C2

##### Material examined.

Morocco – **Middle Atlas Region** • 1♂; Gouffre Izora; 19/V/2022; K. Aattouch, M. El Ouahabi and M.A. El Mouden leg; UAE-FST MA22/24123.

##### North African distribution.

Morocco (El Mouden et al. 2024).

#### ﻿ Rymosia pseudocretensis

Taxon classificationAnimaliaDipteraMycetophilidae

Burghele-Balacesco, 1967

FEB4C734-97D6-5EEC-96C3-327EBE2DC514

##### Material examined.

Morocco – **Middle Atlas Region** • 2♂♂; Gouffre Izora; 19/V/2022; K. Aattouch, M. El Ouahabi and M.A. El Mouden leg; UAE-FST MA22/24124.

##### North African distribution.

Algeria (Burghele-Balacesco 1967); Morocco (Chandler 1994; Chandler et al. 2006; Banamar et al. 2020).

###### ﻿Genus *Stigmatomeria* Tuomikoski, 1966

#### ﻿ Stigmatomeria crassicornis

Taxon classificationAnimaliaDipteraMycetophilidae

(Stannius, 1831)

6C284125-C3A5-5083-868C-3C683043F6CC

##### Material examined.

Morocco – **Middle Atlas Region** • 9♂♂; Gouffre Izora; 19/V/2022; K. Aattouch, M. El Ouahabi and M.A. El Mouden leg; UAE-FST MA22/24125 • 1♂; Grotte Bouslama; 19/V/2022; K. Aattouch, M. El Ouahabi and M.A. El Mouden leg; UAE-FST MA22/24126 • 1♂; Aïn Admam; 22/V/2022; K. Aattouch, M. El Ouahabi and M.A. El Mouden leg; UAE-FST MA22/24127.

##### North African distribution.

Tunisia (unpublished record, leg. H. Malicky, pers. comm. from N. Caspers, 14 April 2004); Morocco (Banamar et al. 2020).

###### ﻿Genus *Synplasta* Skuse, 1890

#### ﻿ Synplasta gracilis

Taxon classificationAnimaliaDipteraMycetophilidae

(Winnertz, 1864)

788EB416-5345-5021-9636-32B4FBE566C9

##### Material examined.

Morocco – **Middle Atlas Region** • 1♂; Gouffre Izora; 19/V/2022; K. Aattouch, M. El Ouahabi and M.A. El Mouden leg; UAE-FST MA22/24128 • 1♂; Grotte Bouslama; 19/V/2022; K. Aattouch, M. El Ouahabi and M.A. El Mouden leg; UAE-FST MA22/24129.

##### North African distribution.

New to Morocco and North Africa.

### ﻿Tribe Mycetophilini Newman, 1834

**Genus *Mycetophila* Meigen, 1803**

#### ﻿ Mycetophila britannica

Taxon classificationAnimaliaDipteraMycetophilidae

Laštovka & Kidd, 1975

CDD81BCB-E5AC-54C6-B335-95EEDD9D436B

##### Material examined.

Morocco – **Middle Atlas Region** • 152♂♂, 215♀♀; Gouffre Izora; 19/V/2022; K. Aattouch, M. El Ouahabi and M.A. El Mouden leg; UAE-FST MA22/24130 • 51♂♂, 60♀♀; Grotte Bouslama; 19/V/2022; K. Aattouch, M. El Ouahabi and M.A. El Mouden leg; UAE-FST MA22/24131 • 5♂♂, 8♀♀; Grotte Bouhayati; 20/V/2022; K. Aattouch, M. El Ouahabi and M.A. El Mouden leg; UAE-FST MA22/24132 • 1♂; Oued Aïn Chabab; 20/V/2022; K. Aattouch, M. El Ouahabi and M.A. El Mouden leg; UAE-FST MA22/24133 • 1♀; Oued Sidi Boulaâla; 20/V/2022; K. Aattouch, M. El Ouahabi and M.A. El Mouden leg; UAE-FST MA22/24134 • 1♂; Forêt Admam; 22/V/2022; K. Aattouch, M. El Ouahabi and M.A. El Mouden leg; UAE-FST MA22/24135.

##### North African distribution.

Morocco (Chandler and Ribeiro 1995; Banamar et al. 2020).

#### ﻿ Mycetophila edwardsi

Taxon classificationAnimaliaDipteraMycetophilidae

Lundström, 1913

F75032C0-65EC-5266-9F97-AEA402DD658D

##### Material examined.

Morocco – **Middle Atlas Region** • 36♂♂; Gouffre Izora; 19/V/2022; K. Aattouch, M. El Ouahabi and M.A. El Mouden leg; UAE-FST MA22/24137 • 11♂♂, 5♀♀; Grotte Bouslama; 19/V/2022; K. Aattouch, M. El Ouahabi and M.A. El Mouden leg; UAE-FST MA22/24138 • 1♂; Forêt Bab Boudir; 20/V/2022; K. Aattouch, M. El Ouahabi and M.A. El Mouden leg; UAE-FST MA22/24136 • 1♂; Forêt Admam; 22/V/2022; K. Aattouch, M. El Ouahabi and M.A. El Mouden leg; UAE-FST MA22/24139.

##### North African distribution.

Morocco (Banamar et al. 2020).

#### ﻿ Mycetophila marginata

Taxon classificationAnimaliaDipteraMycetophilidae

Winnertz, 1864

3156F79D-1ED8-568C-9887-FC70A14EEAEE

##### Material examined.

Morocco – **Middle Atlas Region** • 21♂♂, 51♀♀; Gouffre Izora; 19/V/2022; K. Aattouch, M. El Ouahabi and M.A. El Mouden leg; UAE-FST MA22/24140 • 32♂♂; Grotte Bouslama; 19/V/2022; K. Aattouch, M. El Ouahabi and M.A. El Mouden leg; UAE-FST MA22/24141 • 1♂; Vallée des oiseaux; 19/V/2022; K. Aattouch, M. El Ouahabi and M.A. El Mouden leg; UAE-FST MA22/24142 • 1♂; Grotte Bouhayati; 20/V/2022; K. Aattouch, M. El Ouahabi and M.A. El Mouden leg; UAE-FST MA22/24143.

##### North African distribution.

Morocco (Banamar et al. 2020).

#### ﻿ Mycetophila mitis

Taxon classificationAnimaliaDipteraMycetophilidae

(Johannsen, 1912)

2742D99F-0089-56CB-B3A3-71B5F3A449F0

##### Material examined.

Morocco – **Middle Atlas Region** • 12♂♂; Gouffre Izora; 19/V/2022; K. Aattouch, M. El Ouahabi and M.A. El Mouden leg; UAE-FST MA22/24144 • 3♂♂; Grotte Bouslama; 19/V/2022; K. Aattouch, M. El Ouahabi and M.A. El Mouden leg; UAE-FST MA22/24145 • 2♂♂; Grotte Bouhayati; 20/V/2022; K. Aattouch, M. El Ouahabi and M.A. El Mouden leg; UAE-FST MA22/24146.

##### North African distribution.

New to Morocco and North Africa.

#### ﻿ Mycetophila perpallida

Taxon classificationAnimaliaDipteraMycetophilidae

Chandler, 1993

AD5EEB13-0929-5035-B92B-2020CBB840D5

##### Material examined.

Morocco – **Middle Atlas Region** • 1♂; Forêt Bouhayati; 19/V/2022; K. Aattouch, M. El Ouahabi and M.A. El Mouden leg; UAE-FST MA22/24147 • 16♂♂; Gouffre Izora; 19/V/2022; K. Aattouch, M. El Ouahabi and M.A. El Mouden leg; UAE-FST MA22/24148 • 13♂♂; Grotte Bouslama; 19/V/2022; K. Aattouch, M. El Ouahabi and M.A. El Mouden leg; UAE-FST MA22/24149 • 5♂♂; Grotte Bouhayati; 20/V/2022; K. Aattouch, M. El Ouahabi and M.A. El Mouden leg; UAE-FST MA22/24150.

##### North African distribution.

Cited from North Africa without further details (Chandler 2009); Morocco (Banamar et al 2020).

#### ﻿ Mycetophila pictula

Taxon classificationAnimaliaDipteraMycetophilidae

Meigen, 1830

8D5B82FB-43B2-516A-9298-91420943D50C

##### Material examined.

Morocco – **Middle Atlas Region** • 14♂♂, 20♀♀; Gouffre Izora; 19/V/2022; K. Aattouch, M. El Ouahabi and M.A. El Mouden leg; UAE-FST MA22/24152 • 1♂, 4♀♀; Grotte Bouslama; 19/V/2022; K. Aattouch, M. El Ouahabi and M.A. El Mouden leg; UAE-FST MA22/24153 • 1♀; Forêt Bab Boudir; 20/V/2022; K. Aattouch, M. El Ouahabi and M.A. El Mouden leg; UAE-FST MA22/24151 • 2♂♂, 3♀♀; Grotte Bouhayati; 20/V/2022; K. Aattouch, M. El Ouahabi and M.A. El Mouden leg; UAE-FST MA22/24154.

##### North African distribution.

Morocco (Chandler and Ribeiro 1995; Banamar et al. 2020).

#### ﻿ Mycetophila pumila

Taxon classificationAnimaliaDipteraMycetophilidae

Winnertz, 1864

257632A6-B739-573C-A6CB-76C0D8480DA7

##### Material examined.

Morocco – **Middle Atlas Region** • 1♂, 15♀♀; Gouffre Izora; 19/V/2022; K. Aattouch, M. El Ouahabi and M.A. El Mouden leg; UAE-FST MA22/24156 • 5♂♂; Grotte Bouslama; 19/V/2022; K. Aattouch, M. El Ouahabi and M.A. El Mouden leg; UAE-FST MA22/24157 • 1♂; Vallée des oiseaux; 19/V/2022; K. Aattouch, M. El Ouahabi and M.A. El Mouden leg; UAE-FST MA22/24158; • 1♂; Forêt Bab Boudir; 20/V/2022; K. Aattouch, M. El Ouahabi and M.A. El Mouden leg; UAE-FST MA22/24155.

##### North African distribution.

New to Morocco and North Africa.

#### ﻿ Mycetophila sordida

Taxon classificationAnimaliaDipteraMycetophilidae

van der Wulp, 1874

5500FDB2-B901-582C-AA31-6B897778B881

##### Material examined.

Morocco – **Middle Atlas Region** • 1♂; Grotte Bouslama; 19/V/2022; K. Aattouch, M. El Ouahabi and M.A. El Mouden leg; UAE-FST MA22/24159.

##### North African distribution.

Morocco (Chandler 1994; Banamar et al. 2020).

#### ﻿ Mycetophila spectabilis

Taxon classificationAnimaliaDipteraMycetophilidae

Winnertz, 1864

7F67FDEE-20D3-59B2-BC55-7F7D2AD4914B

##### Material examined.

Morocco – **Middle Atlas Region** • 23♂♂; Gouffre Izora; 19/V/2022; K. Aattouch, M. El Ouahabi and M.A. El Mouden leg; UAE-FST MA22/24160 • 5♂♂; Grotte Bouslama; 19/V/2022; K. Aattouch, M. El Ouahabi and M.A. El Mouden leg; UAE-FST MA22/24161 • 1♂; Grotte Bouhayati; 20/V/2022; K. Aattouch, M. El Ouahabi and M.A. El Mouden leg; UAE-FST MA22/24162 • 1♂; Oued Aïn Chabab; 20/V/2022; K. Aattouch, M. El Ouahabi and M.A. El Mouden leg; UAE-FST MA22/24163 • 1♂; Forêt Admam; 22/V/2022; K. Aattouch, M. El Ouahabi and M.A. El Mouden leg; UAE-FST MA22/24164.

##### North African distribution.

Morocco (Banamar et al. 2020).

#### ﻿ Mycetophila strigatoides

Taxon classificationAnimaliaDipteraMycetophilidae

(Landrock, 1927)

81EC2806-1BCF-521D-A952-CA4AB9437707

##### Material examined.

Morocco – **Middle Atlas Region** • 2♂♂; Gouffre Izora; 19/V/2022; K. Aattouch, M. El Ouahabi and M.A. El Mouden leg; UAE-FST MA22/24165 • 1♂; Grotte Bouslama; 19/V/2022; K. Aattouch, M. El Ouahabi and M.A. El Mouden leg; UAE-FST MA22/24166.

##### North African distribution.

Tunisia (Chandler 1994); Morocco (Banamar et al. 2020).

#### ﻿ Mycetophila unicolor

Taxon classificationAnimaliaDipteraMycetophilidae

Stannius, 1831

41E89201-0835-550E-93EC-3009746D83DD

##### Material examined.

Morocco – **Middle Atlas Region** • 1♂; Grotte Bouslama; 19/V/2022; K. Aattouch, M. El Ouahabi and M.A. El Mouden leg; UAE-FST MA22/24167.

##### North African distribution.

Morocco (Banamar et al. 2020).

#### ﻿ Mycetophila vittipes

Taxon classificationAnimaliaDipteraMycetophilidae

Zetterstedt, 1852

BC6D4C96-EADE-500C-8E11-1ADEC424AD22

##### Material examined.

Morocco – **Middle Atlas Region** • 5♀♀; Gouffre Izora; 19/V/2022; K. Aattouch, M. El Ouahabi and M.A. El Mouden leg; UAE-FST MA22/24169 • 5♂♂, 5♀♀; Grotte Bouslama; 19/V/2022; K. Aattouch, M. El Ouahabi and M.A. El Mouden leg; UAE-FST MA22/24170; • 1♂; Forêt Bab Boudir; 20/V/2022; K. Aattouch, M. El Ouahabi and M.A. El Mouden leg; UAE-FST MA22/24168.

##### North African distribution.

Morocco (Banamar et al. 2020).

###### ﻿Genus *Phronia* Winnertz, 1864

#### ﻿ Phronia biarcuata

Taxon classificationAnimaliaDipteraMycetophilidae

(Becker, 1908)

E4DFDDD8-D1CF-5C58-8501-BD84D10C60A1

##### Material examined.

Morocco – **Middle Atlas Region** • 4♂♂; Gouffre Izora; 19/V/2022; K. Aattouch, M. El Ouahabi and M.A. El Mouden leg; UAE-FST MA22/24171 • 15♂♂; Grotte Bouslama; 19/V/2022; K. Aattouch, M. El Ouahabi and M.A. El Mouden leg; UAE-FST MA22/24172.

##### North African distribution.

Tunisia (Chandler 1994); Morocco (Chandler 1994; Chandler and Ribeiro 1995; Chandler et al. 2006; Banamar et al. 2020).

#### ﻿ Phronia nitidiventris

Taxon classificationAnimaliaDipteraMycetophilidae

(van der Wulp, 1858)

2A77BB9F-3436-54EB-BDE9-3A8DFA6F2740

##### Material examined.

Morocco – **Middle Atlas Region** • 1♂; Grotte Bouslama; 19/V/2022; K. Aattouch, M. El Ouahabi and M.A. El Mouden leg; UAE-FST MA22/24173.

##### North African distribution.

Morocco (Banamar et al. 2020).

#### ﻿ Phronia tenuis

Taxon classificationAnimaliaDipteraMycetophilidae

Winnertz, 1864

8A1C231B-4477-5BB2-AFD4-98E9D5A40FEB

##### Material examined.

Morocco – **Middle Atlas Region** • 9♂♂; Gouffre Izora; 19/V/2022; K. Aattouch, M. El Ouahabi and M.A. El Mouden leg; UAE-FST MA22/24174 • 19♂♂; Grotte Bouslama; 19/V/2022; K. Aattouch, M. El Ouahabi and M.A. El Mouden leg; UAE-FST MA22/24175 • 2♂♂; Aïn Admam; 22/V/2022; K. Aattouch, M. El Ouahabi and M.A. El Mouden leg; UAE-FST MA22/24176 • 2♂♂; Forêt Admam; 22/V/2022; K. Aattouch, M. El Ouahabi and M.A. El Mouden leg; UAE-FST MA22/24177.

##### North African distribution.

Algeria, Tunisia (Chandler 1994); Morocco (Banamar et al. 2020).

#### ﻿ Phronia willistoni

Taxon classificationAnimaliaDipteraMycetophilidae

Dziedzicki, 1889

4CCEA6C9-2E2F-58CB-A273-92EA096FCCDF

##### Material examined.

Morocco – **Middle Atlas Region** • 1♂; Forêt Bab Boudir; 19/V/2022; K. Aattouch, M. El Ouahabi and M.A. El Mouden leg; UAE-FST MA22/24178.

##### North African distribution.

Morocco (Banamar et al. 2020).

###### ﻿Genus *Sceptonia* Winnertz, 1864

#### ﻿ Sceptonia flavipuncta

Taxon classificationAnimaliaDipteraMycetophilidae

Edwards, 1925

424DE52C-042E-5441-B729-F80B5C594DE2

##### Material examined.

Morocco – **Middle Atlas Region** • 1♂; Forêt Admam; 22/V/2022; K. Aattouch, M. El Ouahabi and M.A. El Mouden leg; UAE-FST MA22/24179.

##### North African distribution.

New to Morocco and North Africa.

#### ﻿ Sceptonia intestata

Taxon classificationAnimaliaDipteraMycetophilidae

Plassmann & Schacht, 1990

591C9BD6-AF78-5A46-AA3C-32F2312303B8

##### Material examined.

Morocco – **Middle Atlas Region** • 1♂; Vallée des oiseaux; 19/V/2022; K. Aattouch, M. El Ouahabi and M.A. El Mouden leg; UAE-FST MA22/24180 • 3♂♂; Forêt Admam; 22/V/2022; K. Aattouch, M. El Ouahabi and M.A. El Mouden leg; UAE-FST MA22/24181.

##### North African distribution.

Morocco (Banamar et al. 2020).

#### ﻿ Sceptonia membranacea

Taxon classificationAnimaliaDipteraMycetophilidae

Edwards, 1925

DAB6F5A4-5B32-513B-8047-6F1618648E06

##### Material examined.

Morocco – **Middle Atlas Region** • 1♂; Aïn Admam; 22/V/2022; K. Aattouch, M. El Ouahabi and M.A. El Mouden leg; UAE-FST MA22/24182 • 2♂♂; Forêt Admam; 22/V/2022; K. Aattouch, M. El Ouahabi and M.A. El Mouden leg; UAE-FST MA22/24183.

##### North African distribution.

Morocco (Banamar et al. 2020).

###### ﻿Genus *Trichonta* Winnertz, 1864

#### ﻿ Trichonta apicalis

Taxon classificationAnimaliaDipteraMycetophilidae

Strobl 1897

66492DBB-E11E-5597-A838-5B7E84493959

##### Material examined.

Morocco – **Middle Atlas Region** • 1♂; Grotte Bouslama; 19/V/2022; K. Aattouch, M. El Ouahabi and M.A. El Mouden leg; UAE-FST MA22/24184.

##### North African distribution.

New to Morocco and North Africa.

#### ﻿ Trichonta clavigera

Taxon classificationAnimaliaDipteraMycetophilidae

Lundström, 1913

0C591881-A275-543A-A10D-B5F9D3F58B9E

##### Material examined.

Morocco – **Middle Atlas Region** • 1♂; Grotte Bouslama; 19/V/2022; K. Aattouch, M. El Ouahabi and M.A. El Mouden leg; UAE-FST MA22/24185.

##### North African distribution.

Tunisia (unpublished record, leg. H. Malicky, pers. comm. from N. Caspers, 14 April 2004); new to Morocco.

#### ﻿ Trichonta foeda

Taxon classificationAnimaliaDipteraMycetophilidae

Loew, 1869

8ABA54AC-2798-5DDB-92E2-4066C80FB16C

##### Material examined.

Morocco – **Middle Atlas Region** • 2♂♂; Grotte Bouslama; 19/V/2022; K. Aattouch, M. El Ouahabi and M.A. El Mouden leg; UAE-FST MA22/24186 • 1♂; Gouffre Izora; 19/V/2022; K. Aattouch, M. El Ouahabi and M.A. El Mouden leg; UAE-FST MA22/24187.

##### North African distribution.

Morocco (Banamar et al. 2020).

#### ﻿ Trichonta vitta

Taxon classificationAnimaliaDipteraMycetophilidae

(Meigen, 1830)

36D3F807-B5D8-571B-8F58-18963C930F43

##### Material examined.

Morocco – **Middle Atlas Region** • 5♂♂; Gouffre Izora; 19/V/2022; K. Aattouch, M. El Ouahabi and M.A. El Mouden leg; UAE-FST MA22/24188 • 3♂♂; Grotte Bouslama; 19/V/2022; K. Aattouch, M. El Ouahabi and M.A. El Mouden leg; UAE-FST MA22/24189 • 1♂; Grotte Bouhayati; 20/V/2022; K. Aattouch, M. El Ouahabi and M.A. El Mouden leg; UAE-FST MA22/24190 • 3♂♂; Oued Aïn Chabab; 20/V/2022; K. Aattouch, M. El Ouahabi and M.A. El Mouden leg; UAE-FST MA22/24191 • 1♂; Forêt Admam; 22/V/2022; K. Aattouch, M. El Ouahabi and M.A. El Mouden leg; UAE-FST MA22/24192.

##### North African distribution.

Algeria (Gagné 1981; Hackman et al. 1988; Chandler 1994) ; Morocco (Banamar et al. 2020).

###### ﻿Genus *Zygomyia* Winnertz, 1864

#### ﻿ Zygomyia humeralis

Taxon classificationAnimaliaDipteraMycetophilidae

(Wiedemann, 1817)

B5C6A89C-5874-5E72-A69B-6667263924E2

##### Material examined.

Morocco – **Middle Atlas Region** • 8♂♂; Gouffre Izora; 19/V/2022; K. Aattouch, M. El Ouahabi and M.A. El Mouden leg; UAE-FST MA22/24193 • 1♂; Grotte Bouslama; 19/V/2022; K. Aattouch, M. El Ouahabi and M.A. El Mouden leg; UAE-FST MA22/24194 • 1♂; Aïn Admam; 22/V/2022; K. Aattouch, M. El Ouahabi and M.A. El Mouden leg; UAE-FST MA22/24195 • 1♂; Forêt Admam; 22/V/2022; K. Aattouch, M. El Ouahabi and M.A. El Mouden leg; UAE-FST MA22/24196.

##### North African distribution.

Tunisia (unpublished record, leg. H. Malicky, pers. comm. from N. Caspers, 14 April 2004); Morocco (Banamar et al. 2020).

#### ﻿ Zygomyia valeriae

Taxon classificationAnimaliaDipteraMycetophilidae

Chandler, 1991

D87420E2-CF74-501D-B426-284D737EF060

##### Material examined.

Morocco – **Middle Atlas Region** • 3♂♂; Grotte Bouslama; 19/V/2022; K. Aattouch, M. El Ouahabi and M.A. El Mouden leg; UAE-FST MA22/24197.

##### North African distribution.

New to Morocco and North Africa.

#### ﻿ Zygomyia valida

Taxon classificationAnimaliaDipteraMycetophilidae

Winnertz, 1864

86B0C972-11FE-55AD-A1C8-5327AD53880A

##### Material examined.

Morocco – **Middle Atlas Region** • 11♂♂; Gouffre Izora; 19/V/2022; K. Aattouch, M. El Ouahabi and M.A. El Mouden leg; UAE-FST MA22/24198 • 4♂♂; Grotte Bouslama; 19/V/2022; K. Aattouch, M. El Ouahabi and M.A. El Mouden leg; UAE-FST MA22/24199 • 1♂; Grotte Bouhayati; 20/V/2022; K. Aattouch, M. El Ouahabi and M.A. El Mouden leg; UAE-FST MA22/24200.

##### North African distribution.

Morocco (Banamar et al. 2020).

### ﻿Subfamily Mycomyinae Edwards,1925

**Genus *Mycomya* Rondani, 185**

#### ﻿ Mycomya prominens

Taxon classificationAnimaliaDipteraMycetophilidae

(Lundström, 1913)

ACD8DE47-B089-5E5E-BC23-E80FE4031553

##### Material examined.

Morocco – **Middle Atlas Region** • 1♂; Gouffre Izora; 19/V/2022; K. Aattouch, M. El Ouahabi and M.A. El Mouden leg; UAE-FST MA22/24201 • 3♂♂; Oued Aïn Chabab; 20/V/2022; K. Aattouch, M. El Ouahabi and M.A. El Mouden leg; UAE-FST MA22/24202.

##### North African distribution.

Morocco (El Mouden et al. 2024).

#### ﻿ Mycomya pygmalion

Taxon classificationAnimaliaDipteraMycetophilidae

Väisänen, 1984

4DB20FB4-910E-5446-A92D-FC72AA8FC3F8

##### Material examined.

Morocco – **Middle Atlas Region** • 1♂; Grotte Bouhayati; 20/V/2022; K. Aattouch, M. El Ouahabi and M.A. El Mouden leg; UAE-FST MA22/24203.

##### North African distribution.

Morocco (Banamar et al. 2020).

#### ﻿ Mycomya tenuis

Taxon classificationAnimaliaDipteraMycetophilidae

(Walker, 1856)

9918E0CF-7B14-5583-9F49-27A522286138

##### Material examined.

Morocco – **Middle Atlas Region** • 1♂; Forêt Admam; 22/V/2022; K. Aattouch, M. El Ouahabi and M.A. El Mouden leg; UAE-FST MA22/24204.

##### North African distribution.

New to Morocco and North Africa.

#### ﻿ Mycomya tumida

Taxon classificationAnimaliaDipteraMycetophilidae

(Winnertz, 1864)

9BB44DF7-C4F5-5F38-86EB-9E3F38E48ACD

##### Material examined.

Morocco – **Middle Atlas Region** • 1♂; Oued Taourirt; 20/V/2022; K. Aattouch, M. El Ouahabi and M.A. El Mouden leg; UAE-FST MA22/24205.

##### North African distribution.

Morocco (Banamar et al. 2020).

### ﻿Subfamily Sciophilinae Rondani, 1840

**Genus *Azana* Walker, 1856**

#### ﻿ Azana anomala

Taxon classificationAnimaliaDipteraMycetophilidae

Staeger, 1840

DEB9BEEB-43EC-5B8F-9049-1904E1EA7C54

##### Material examined.

Morocco – **Middle Atlas Region** • 6♂♂; Forêt Bouhayati; 19/V/2022; K. Aattouch, M. El Ouahabi and M.A. El Mouden leg; UAE-FST MA22/24206 • 1♂; Forêt gouffre Friouato; 19/V/2022; K. Aattouch, M. El Ouahabi and M.A. El Mouden leg; UAE-FST MA22/24207 • 2♂♂; Grotte Bouslama; 19/V/2022; K. Aattouch, M. El Ouahabi and M.A. El Mouden leg; UAE-FST MA22/24208 • 1♂; Village Bab Boudir; 19/V/2022; K. Aattouch, M. El Ouahabi and M.A. El Mouden leg; UAE-FST MA22/24209 • 1♂; Oued Taourirt; 20/V/2022; K. Aattouch, M. El Ouahabi and M.A. El Mouden leg; UAE-FST MA22/24210 • 1♂; Aïn Admam; 22/V/2022; K. Aattouch, M. El Ouahabi and M.A. El Mouden leg; UAE-FST MA22/24211 • 1♂; Forêt Admam; 22/V/2022; K. Aattouch, M. El Ouahabi and M.A. El Mouden leg; UAE-FST MA22/24212.

##### North African distribution.

Algeria (Becker 1907 as *A.altera*; Hackman et al. 1988); Morocco (Banamar et al. 2020).

###### ﻿Genus *Sciophila* Meigen, 1818

#### ﻿ Sciophila geniculata

Taxon classificationAnimaliaDipteraMycetophilidae

Zetterstedt, 1838

E7CAE327-73C3-5601-9087-D5D50125BF07

##### Material examined.

Morocco – **Middle Atlas Region** • 1♂; Gouffre Izora; 19/V/2022; K. Aattouch, M. El Ouahabi and M.A. El Mouden leg; UAE-FST MA22/24213.

##### North African distribution.

New to Morocco and North Africa.

#### ﻿ Sciophila insolita

Taxon classificationAnimaliaDipteraMycetophilidae

Santos Abreu, 1920

03DA21F9-4142-515C-A427-1D5F147F41C2

##### Material examined.

Morocco – **Middle Atlas Region** • 1♂; Gouffre Izora; 19/V/2022; K. Aattouch, M. El Ouahabi and M.A. El Mouden leg; UAE-FST MA22/24214.

##### North African distribution.

New to Morocco and North Africa.

#### ﻿ Sciophila parviareolata

Taxon classificationAnimaliaDipteraMycetophilidae

Santos Abreu, 1920

A5416582-F28F-505D-88C6-9640CE260583

##### Material examined.

Morocco – **Middle Atlas Region** • 1♂; Grotte Bouslama; 19/V/2022; K. Aattouch, M. El Ouahabi and M.A. El Mouden leg; UAE-FST MA22/24215 • 1♂; Vallée des oiseaux; 19/V/2022; K. Aattouch, M. El Ouahabi and M.A. El Mouden leg; UAE-FST MA22/24216 • 1♂; Grotte Bouhayati; 20/V/2022; K. Aattouch, M. El Ouahabi and M.A. El Mouden leg; UAE-FST MA22/24217.

##### North African distribution.

New to Morocco and North Africa.

## ﻿Discussion

### ﻿Fungus gnats of North Africa in numbers

The newly recorded species from Morocco have raised the total number of North African fungus gnat species from 114 to 133. While the number of Bolitophilidae species remains unchanged, one new species has been added to the Keroplatidae family, bringing the total to 20 species. The Mycetophilidae family has seen the most significant increase, growing from 94 to 112 species, and now includes an additional genus, making a total of 31 genera (Table 2).

**Table 2.** Summary of Bolitophilidae, Keroplatidae, and Mycetophilidae species and genera recorded from the North African countries.

	Bolitophilidae		Keroplatidae		Mycetophilidae		Total	
	Species	Genera	Species	Genera	Species	Genera	Species	Genera
North Africa	1	1	20	10	112	31	133	42
Morocco	1	1	13	7	94	30	108	38
Algeria	1	1	5	3	23	13	29	17
Tunisia	0	0	9	7	25	16	34	23
Libya	0	0	0	0	0	0	0	0
Egypt	0	0	2	1	0	0	2	1

Morocco, with the greatest number of total species, has more than 80% of the North African fungus gnats fauna (Table 2). In contrast, neighbouring countries such as Algeria and Tunisia account for only 20% and 25% of the total regional species, respectively, based on non-systematic study data. Interestingly, there is no published or examined collection data available for the fungus gnats of Libya, while Egypt has only two recorded Keroplatidae species.

However, the similar climates of Morocco, Algeria, and Tunisia, with their comparable geological compositions, such as the extension of the High Atlas Mountains into Algeria and Tunisia, suggest that more species of fungus gnats may be found in these regions. By any manner, given the limited research and knowledge about fungus gnats in other North African countries, this part of the discussion serves as a preliminary comparison of species presence and richness across the region.

### ﻿Fungus gnat distribution relative to surrounding areas

It is striking that all the previously described species that are newly recorded from Morocco have a widespread Palearctic distribution. Although, the influence of Western European distribution and Mediterranean distribution seems to have an equal impact since all of the species newly cited in our study are in common with Western Europe and with the Mediterranean region. As usual, faunistic exchanges with the Iberian Peninsula are always higher, as much as in this current study, emphasising that more than 63% of the 18 species newly recorded for Morocco in this study are in common with those of the Iberian Peninsula (Chandler and Báez 2002). Approximately 26% of the species are shared with the North Atlantic islands’ region, a noteworthy figure considering the significant role of endemism in shaping the fauna of these islands As for North Africa, only one species newly recorded from Morocco seems to be in common with the rest of the area, which can be explained by the lack of comparable studies in other North African countries. Finally, *Docosiatazekkae* Chandler, El Mouden & Belqat, **sp. nov.** described in this paper, in addition to the other five Mycetophilidae species recently described in El Mouden et al. (2024), raise the percentage of the species of fungus gnat recorded only from Morocco so far (Mycetophilidae family particularly) from 0% to 5.5% (El Mouden et al. 2024). This percentage is considered to be highly important, and it highlights the potential of finding more species of fungus gnats new to science from Morocco.

### ﻿Fungus gnats of the caves

Several species of fungus gnats are known to inhabit cavities and especially cave entrances for the perfect climatic conditions they offer which correspond to the optimum life conditions for this dipteran group of insects. The results of our study pointed in the same direction since 55 species of fungus gnats of the 71 species recorded were found in the three cave entrances we managed to sample: Grotte Bousslama, Gouffre Izora, and Grotte Bouhayati, which are all located in the PNTZK. The first cave, Grotte Bousslama, is situated in the heart of a dense forest, a natural, well-preserved area. While Gouffre Izora is positioned near a managed rest area, one of the few well-maintained rest areas of the PNTZK, it still belongs to a forested habitat with a high level of protection. The entrances to the two caves are sheltered by a significant vegetative cover, which makes them the perfect ecosystem for the fungus gnats; this is supported by the high number of findings in each locality: 45 species from 16 genera were present at Grotte Bousslama, while 41 species from 18 genera were found to inhabit Gouffre Izora. The third cave, Grotte Bouhayati, is generally a natural hole in the ground under the limit of a forest area devoid of vegetation at its entrance, where a total of 21 species from nine genera were collected from this cave. Most of these species are found also in other types of habitats within the park, while a total of 26 species were found nowhere else but in caves. In other words, more than one-third (37%) of the PNTZK fungus gnat species were recorded only in caves.

However, most of these species are not primarily cave dwellers. According to literature records from other geographical areas and to the former survey on Mycetophilidae of Morocco by Banamar et al. (2020), they have been found in other different biotopes. Members of this family are well known to enter caves for aestivation or hibernation. We believe that the reason for this marked effect on our results was the prevailing climate. During the period of sampling in late May, the temperature was quite high, commonly between 27 °C and 37 °C. Consequently, the majority of fungus gnats seek refuges like these cavities when external temperatures are high to shelter from the heat.

## ﻿Conclusions

This study contributes significantly to our knowledge of the biodiversity of the fungus gnats known from Morocco and North Africa. Two genera and 19 species were recorded for the first time from Morocco which increases the number of species in the fungus gnat fauna from 89 to 108. As for North Africa, 18 of these species and one genus are considered new to the region so the fungus gnat species number jumps from 114 to 133. Also, the discovery of the new *Docosia* species from Morocco together with the five mycetophilid species previously described by El Mouden et al. (2024), highlights biogeographic and conservation interest areas of the studied areas. If this indicates anything, it is that the North African region still hides more secrets about the fungus gnats than it shows, either as new records or even as species new to science. This should stimulate the appetite for more hard work and more profound studies in every country of the region to lead to curtain raising on the discoveries that are waiting for us.

## Supplementary Material

XML Treatment for Bolitophila saundersii

XML Treatment for Macrocera fasciata

XML Treatment for Neoplatyura biumbrata

XML Treatment for Orfelia fasciata

XML Treatment for Boletina lundstroemi

XML Treatment for Coelosia fusca

XML Treatment for Docosia gilvipes

XML Treatment for Docosia melita

XML Treatment for Docosia tazekkae

XML Treatment for Megophthalmidia amsemlili

XML Treatment for Synapha fasciata

XML Treatment for Tetragoneura

XML Treatment for Leia arcana

XML Treatment for Leia beckeri

XML Treatment for Leia bimaculata

XML Treatment for Novakia scatopsiformis

XML Treatment for Novakia simillima

XML Treatment for Allodia ornaticollis

XML Treatment for Brevicornu fissicauda

XML Treatment for Brevicornu griseicolle

XML Treatment for Brevicornu intermedium

XML Treatment for Brevicornu sericoma

XML Treatment for Brevicornu verralli

XML Treatment for Cordyla crassicornis

XML Treatment for Cordyla murina

XML Treatment for Exechia dorsalis

XML Treatment for Exechia fulva

XML Treatment for Exechia fusca

XML Treatment for Exechia spinuligera

XML Treatment for Exechia neorepanda

XML Treatment for Pseudexechia trivittata

XML Treatment for Pseudexechia tuomikoskii

XML Treatment for Rymosia beaucournui

XML Treatment for Rymosia ebejeri

XML Treatment for Rymosia pseudocretensis

XML Treatment for Stigmatomeria crassicornis

XML Treatment for Synplasta gracilis

XML Treatment for Mycetophila britannica

XML Treatment for Mycetophila edwardsi

XML Treatment for Mycetophila marginata

XML Treatment for Mycetophila mitis

XML Treatment for Mycetophila perpallida

XML Treatment for Mycetophila pictula

XML Treatment for Mycetophila pumila

XML Treatment for Mycetophila sordida

XML Treatment for Mycetophila spectabilis

XML Treatment for Mycetophila strigatoides

XML Treatment for Mycetophila unicolor

XML Treatment for Mycetophila vittipes

XML Treatment for Phronia biarcuata

XML Treatment for Phronia nitidiventris

XML Treatment for Phronia tenuis

XML Treatment for Phronia willistoni

XML Treatment for Sceptonia flavipuncta

XML Treatment for Sceptonia intestata

XML Treatment for Sceptonia membranacea

XML Treatment for Trichonta apicalis

XML Treatment for Trichonta clavigera

XML Treatment for Trichonta foeda

XML Treatment for Trichonta vitta

XML Treatment for Zygomyia humeralis

XML Treatment for Zygomyia valeriae

XML Treatment for Zygomyia valida

XML Treatment for Mycomya prominens

XML Treatment for Mycomya pygmalion

XML Treatment for Mycomya tenuis

XML Treatment for Mycomya tumida

XML Treatment for Azana anomala

XML Treatment for Sciophila geniculata

XML Treatment for Sciophila insolita

XML Treatment for Sciophila parviareolata

## ﻿Additional information

### Author ORCIDs

Mohamed Amin El Mouden https://orcid.org/0009-0002-6085-924X

Peter J. Chandler https://orcid.org/0009-0000-4789-3596

Imane Saidoun https://orcid.org/0009-0005-2809-5384

Ouafaa Driauach https://orcid.org/0000-0002-9748-2450

Boutaïna Belqat https://orcid.org/0000-0003-2857-7699

### Data availability

All of the data that support the findings of this study are available in the main text.
